# Growth, Toxin Production and Allelopathic Effects of *Pseudo-nitzschia multiseries* under Iron-Enriched Conditions

**DOI:** 10.3390/md15100331

**Published:** 2017-10-24

**Authors:** Bruna Fernanda Sobrinho, Luana Mocelin de Camargo, Leonardo Sandrini-Neto, Cristian Rafael Kleemann, Eunice da Costa Machado, Luiz Laureno Mafra

**Affiliations:** 1 Centro de Estudos do Mar, Universidade Federal do Paraná, P.O. Box 1, Pontal do Paraná 83255-976, PR, Brazil; brunafernanda.sob@gmail.com (B.F.S.); lumocelin@gmail.com (L.M.d.C.); leonardosandrini@gmail.com (L.S.-N.); 2 Instituto Federal de Santa Catarina, Av. Abraão João Francisco, 3899, Itajaí 88307-303, SC, Brazil; cristian.kleemann@gmail.com; 3 Instituto de Oceanografia, Universidade Federal do Rio Grande, Av. Itália, km 8, Carreiros, Rio Grande 96203-900, RS, Brazil; eunice.ufpr@gmail.com

**Keywords:** harmful algae, toxic diatoms, domoic acid, phycotoxins, allelopathy, iron fertilization

## Abstract

In order to assess the effects of Fe-enrichment on the growth and domoic acid (DA) production of the toxigenic diatom *Pseudo-nitzschia multiseries*, static cultures that received the addition of different iron (Fe) concentrations were maintained for 30 days. Intra- and extracellular DA concentrations were evaluated over time, and growth and chain-formation were compared to those of non-toxic diatoms, *Bacillaria* sp. Growth rates of *P. multiseries* (μ = 0.45–0.73 d^−1^) were similar among cultures containing different Fe concentrations. Likewise, the similar incidence and length of *P. multiseries* stepped cell chains (usually 2–4; up to 8-cell long) among the treatments reinforces that the cultures were not growth-inhibited under any condition tested, suggesting an efficient Fe acquisition mechanism. Moreover, DA concentrations were significantly higher under the highest Fe concentration, indicating that Fe is required for toxin synthesis. *Bacillaria* sp. reached comparable growth rates under the same Fe concentrations, except when the dissolved cell contents from a *P. multiseries* culture was added. The 50–70% reduction in cell density and 70–90% decrease in total chlorophyll-*a* content of *Bacillaria* sp. at early stationary growth phase indicates, for the first time, an allelopathic effect of undetermined compounds released by *Pseudo-nitzschia* to another diatom species.

## 1. Introduction

The first episode of human intoxication associated with compounds produced by diatoms was recorded in 1987 in Cardigan Bay estuary, Prince Edward Island, Canada [1]. At that time, a bloom of the pennate diatom *Pseudo-nitzschia multiseries* caused the hospitalization of >100 people (4 deaths) who had consumed mussels contaminated with a neurotoxin identified as domoic acid (DA) [1,2]. Since then, anomalously high DA concentrations have been related to mortalities of the marine fauna in different coastal regions (e.g., [3,4,5]).

The neurotoxin DA is the causative agent of amnesic shellfish poisoning (ASP) in humans upon consumption of contaminated shellfish [6]. Although outbreaks of human poisoning by DA are not commonplace, many contamination incidents might not be adequately reported. The symptomatology and the rapid elimination of DA from the body make it difficult to clinically verify the intoxication [7]. During a bloom of *Pseudo-nitzschia australis* in 2014 at Galician coastal waters (Spain), for instance, two people reported symptoms of ASP (confusion and memory loss) after consumption of cooked mussels [8].

*Pseudo-nitzschia* spp. are common and abundant components of the coastal phytoplankton assemblage across the globe [9]. Although many authors (e.g., Wells et al. [10]) believe that all *Pseudo-nitzschia* species could become toxigenic under specific environmental conditions, only 26 out of ~45 described *Pseudo-nitzschia* species have been confirmed as DA producers so far [11]. Toxin production is greatly variable, depending on the species, strain, and physiological condition of the population [12,13]. Hence, the occurrence of *Pseudo-nitzschia* spp. at moderate to high cell abundance is not always coupled with the contamination of filter-feeding organisms with unsafe DA levels (e.g., Mafra et al. [14]).

As assessed by both field and laboratory studies, several environmental variables may stimulate DA production by these diatoms, including changes in the photoperiod [15,16], pH [17], salinity [18], temperature [19], and the availability of macronutrients, like silicon (Si), phosphorus (P) [20,21] and nitrogen (N) [22,23], and micronutrients such as copper (Cu) and iron (Fe) [6,10,24,25,26].

Iron is an essential trace element to the metabolism of primary producers, critical in the processes of photosynthesis, respiration, nitrogen reduction and fixation [27]. In marine environments, the fraction of Fe available for biological incorporation varies according to the dominant chemical forms, the preferences of phytoplankton cells for some of these, the balance between the reaction kinetics of Fe exchange among chemical species, and the mechanisms of Fe capture, specific to each species of phytoplankton [28]. According to the on-deck incubation experiments by Endo et al. [29], the addition of Fe would induce the formation of diatom blooms, demonstrating that growth of diatoms can be eventually limited by low Fe availability in seawater.

One of the possible ecological roles of DA is to chelate micronutrients like Fe and Cu [6,10,25], as suggested by the presence of three carboxyl groups in its chemical structure [24]. At high concentrations, DA is able to compete with other organic binders for the capture of dissolved Fe [6]. At lower levels, the toxin facilitates micronutrient absorption by increasing the exchange rates between ligands in solution and on the cell surface [30]. Thus, DA-producing diatoms might have developed more effective strategies for the acquisition of dissolved Fe relative to other phytoplankton species [10], enabling them to grow efficiently in seawater even under limiting conditions of this important micronutrient [6]. However, several aspects related to the mechanisms of DA synthesis, as well as the possible role of DA in conferring *P. multiseries* a competitive advantage under limiting Fe concentrations, must be better understood in order to assess the real ecological relevance of this toxin.

The present study combines a series of laboratory experiments with the aim of evaluating (i) the influence of increasing bioavailable Fe concentrations on the growth and toxin production by *P. multiseries*; and (ii) the possible role of DA in facilitating Fe acquisition by both *P. multiseries* and a non-toxigenic, chain former pennate diatom, *Bacillaria* sp. (Bacillariaceae), under low Fe concentrations. The possible negative effects of compounds produced by *P. multiseries* (i.e., allelopathy) on *Bacillaria* sp. growth were additionally assessed. Cell abundance, chain formation, and intra- and extracellular toxin levels were quantified over time in static cultures, and compared across the different experimental treatments.

## 2. Results

### 2.1. Experiment 1: Pseudo-nitzschia multiseries

#### 2.1.1. Growth

**Figure 1:**
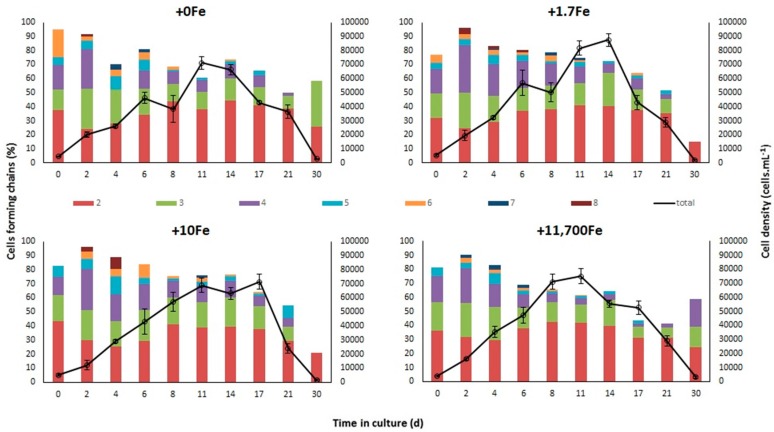
Average (±standard error) cell density (solid line, *n* = 4) and percentage of cells forming chains of 2 to 8 cells throughout the growth cycle of *Pseudo-nitzschia multiseries* in static cultures that received the addition of distinct iron concentrations: +0Fe; +1.7Fe; +10Fe; and +11,700Fe nmol·L^−1^. Colors of the stacked bars represent different cell chain lengths.

The exponential growth rate of *P. multiseries*, measured between days 0 and 8 of the experiment (µ_0–8_, avg ± std) was higher in the +11,700 nmol Fe·L^−1^ (“+11,700Fe”) treatment (µ_0–8_ = 0.36 ± 0.02d^−1^) compared to all other treatments (µ_0–8_ = 0.25–0.31) (Table 1). Conversely, cultures reached greater maximum cell densities in the +1.7Fe treatment (87,412 ± 8592 cell·mL^−1^ on the 14th day) relative to the other treatments, where *P. multiseries* attained from 71,212 ± 9132 cell·mL^−1^ (in +0Fe) to 74,962 ± 10,699 cell·mL^−1^ (in +11,700Fe) after 11–17 days (Figure 1). For both growth rate and cell density, however, average values were not significantly different among treatments (see Supplementary Table S1 for statistics).

In all treatments, *P. multiseries* formed linear chains of up to eight cells. Both the percentage of cells forming chains and the average number of cells per chain decreased gradually throughout the growth cycle, with no significant differences among the experimental treatments. In general, short chains with 2–4 cells predominated in all cultures (Figure 1).

**Figure 2:**
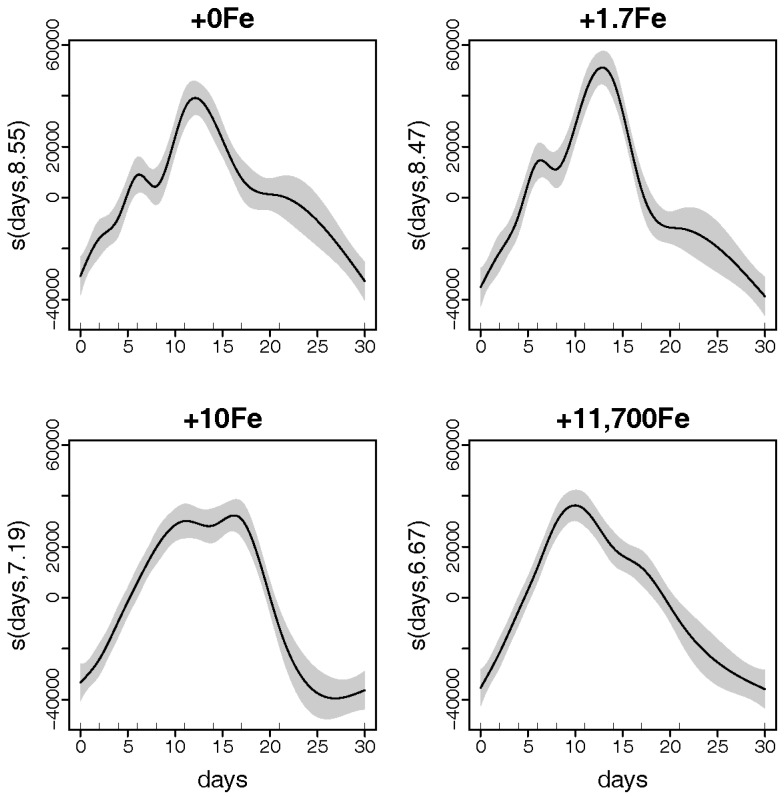
Estimated smoothing curves showing the relationship (solid line) between *Pseudo-nitzschia multiseries* densities and days for each Fe-enrichment treatment level: +0Fe; +1.7Fe; +10Fe; and +11,700Fe nmol·L^−1^. Shaded area represents the standard error of the smooth curve. The rug plots on the *x*-axis indicate the days measurements were taken. Numbers after the variable name on the *y*-axis represent estimated degrees of freedom.

There was a very strong relationship between *P. multiseries* cell densities and experiment days for each Fe addition treatment (Deviance explained = 92.7%; Figure 2). Expected densities from generalized additive models (GAMs) reached maximum values at ~11 days after the beginning of the experiment in all treatments. However, the shapes of the smoother curves as well as the maximum expected density values varied among treatments. Expected cell densities were significantly higher in the +1.7Fe treatment (Figure 2) than in other treatments receiving the addition of different Fe concentrations.

#### 2.1.2. Domoic Acid

The intracellular, particulate DA concentrations (pDA) increased significantly over time (*p* = 2.2 × 10^−16^), and were significantly affected by the experimental treatments (*p* = 4.5 × 10^−4^). Greater pDA values were measured upon the highest addition of Fe (11,700 µmol·L^−1^) compared to the other treatments, as recorded on days 8 and 17 (0.20–0.45 vs. 0.03–0.27 pg·cell^−1^, respectively), and especially on day 30, when the highest intracellular levels were reported in all treatments (2.07 pg·cell^−1^ in +11,700Fe vs. 0.84–1.29 pg·cell^−1^ in the other treatments) (see Supplementary Table S2 for statistical results).

The extracellular, dissolved DA concentrations (dDA), also increased significantly along the growth curve (*p* = 5.4 × 10^−9^), but, in general, did not vary consistently across the experimental treatments (*p* = 0.39) when accounted on a per cell basis. At exponential growth (day 8), the treatment without Fe addition yielded a higher mean dDA value (1.85 pg·cell^−1^) relative to the other treatments (0.55–0.81 pg·cell^−1^). In contrast, during the stationary phase (day 17), the highest dDA concentrations were measured in the treatment that received the highest Fe addition (91.3 vs. 1.95–25.8 pg·cell^−1^). Later on, during the decline phase (day 30), the highest dDA values were again detected in the treatment without Fe addition (18.6 vs. 4.56–7.24 pg·cell^−1^) (Table 2). Concentrations of dDA were greater on day 17 compared to all other sampling days (Supplementary Table S2).

### 2.2. Experiment 2: Bacillaria *sp*.

#### 2.2.1. Growth

**Figure 3:**
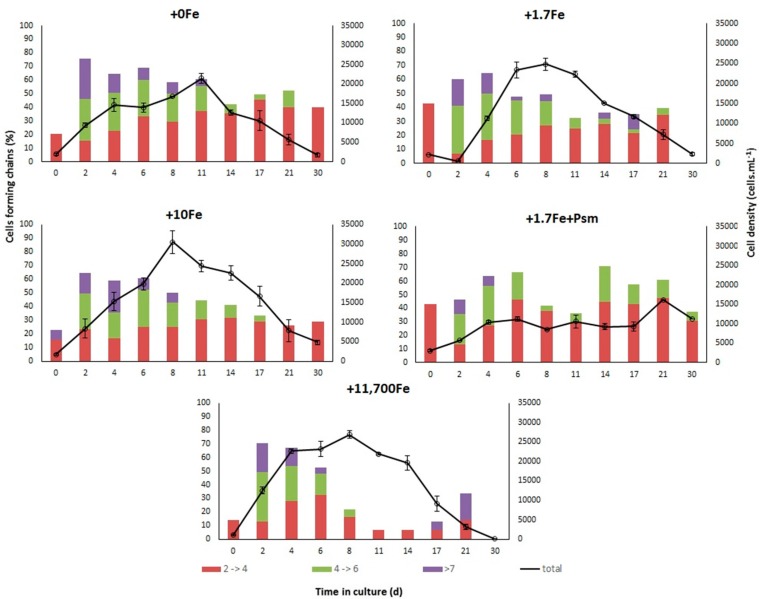
Average (±standard error) cell density (solid line, *n* = 3) and percentage of cells forming chains throughout the growth cycle of *Bacillaria* sp. in static cultures that received the addition of distinct iron concentrations: +0Fe; +1.7Fe; +10Fe; and +11,700Fe nmol·L^−1^. An extra treatment (+1.7Fe+Psm) was created with the addition of 1.7 nmol Fe·L^−1^ and the dissolved cell contents from a *P. multiseries* culture containing 52 ng DA·mL^−1^ and other undetermined compounds. Colors of the stacked bars represent different cell chain lengths.

*Bacillaria* sp. attained up to 30,500 ± 5007 cell·mL^−1^ in treatment +10Fe, 26,850 ± 2333 cell·mL^−1^ in treatment +11,700Fe, 24,750 ± 3606 cell·mL^−1^ in +1.7Fe, all on the 8th day of culture, and only 21,500 ± 2186 cell·mL^−1^ on the 11th day in the treatment with no Fe addition (Figure 3). Interestingly, the maximum cell density was even lower in the treatment that received the dissolved cell contents from a *P. multiseries* culture (+1.7Fe+Psm), attaining only 16,200 cell·mL^−1^ after 21 days in culture. Maximum cell densities were different among treatments (*p* = 0.018; one-way ANOVA), probably due to the lower values measured in +1.7Fe+Psm, although the Student–Newman–Keuls (SNK) post-hoc test was not able to detect that (Supplementary Table S1). The highest exponential growth rate between days 0 and 8 (µ_0–8_ = 0.41 ± 0.05 d^−1^) was measured in the treatment that received the greatest Fe addition (+11,700Fe). Within this same culture period, growth rates were significantly lower (*p* = 0.0006; one-way ANOVA) for the +1.7Fe+Psm treatment (µ_0–8_ = 0.13 ± 0.02 d^−1^), and ranged from 0.28 to 0.36 d^−1^ for the other treatments (Table 1 and Table S1).

Surprisingly, the maximum growth rates of *Bacillaria* sp. (µ = 0.80–1.25 d^−1^) were consistently higher than those calculated for *P. multiseries* (µ = 0.54–0.74 d^−1^) in cultures with the addition of equivalent Fe concentrations, except for the +1.7Fe+Psm treatment (µ = 0.36 d^−1^) (Table 1). In all treatments, *Bacillaria* sp. formed chains, some containing more than 8 cells. However, there was a predominance of short chains, with 2 to 4 cells (Figure 3), similar to what was observed for *P. multiseries*.

**Figure 4:**
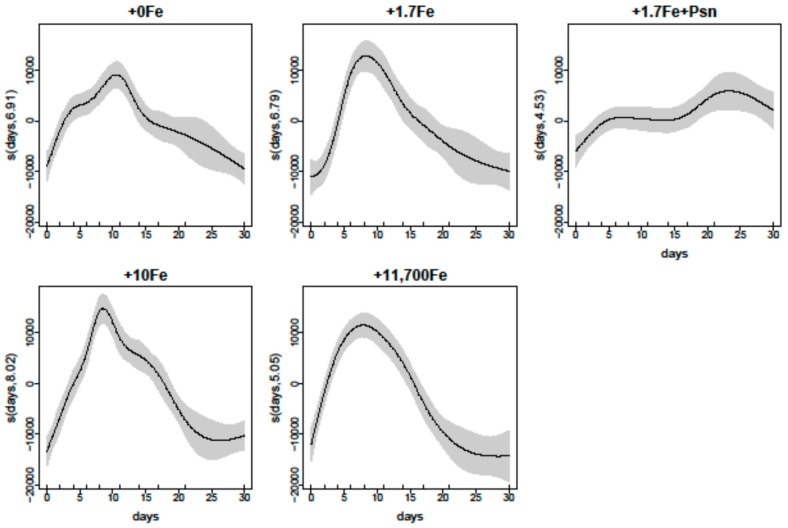
Estimated smoothing curves showing the relationship (solid line) between *Bacillaria* sp. densities and days for each Fe-enrichment treatment level: +0Fe; +1.7Fe; +10Fe; and +11,700Fe nmol·L^−1^, and an extra treatment (+1.7Fe+Psm) with the addition of 1.7 nmol Fe·L^−1^ plus the dissolved cell contents from a culture of *P. multiseries* containing domoic acid at 52 ng·mL^−1^ and other undetermined compounds. Shaded area represents the standard error of the smooth curve. The rug plots on the *x*-axis indicate the days measurements were taken. Numbers after the variable name on the *y*-axis represent estimated degrees of freedom.

Similarly to *P. multiseries*, the relationship between *Bacillaria* sp. cell densities and experiment days for each Fe addition treatment was very strong (Deviance explained = 92%; Figure 4). Expected densities reached maximum values at 8–11 days after the start of the experiment in all Fe treatments, except for the +1.7Fe+Psm treatment, where growth inhibition was observed at early stages. Shapes of the smoother curves, as well as the maximum expected density values, varied greatly among treatments (Figure 4).

#### 2.2.2. Chlorophyll-*a*

**Figure 5:**
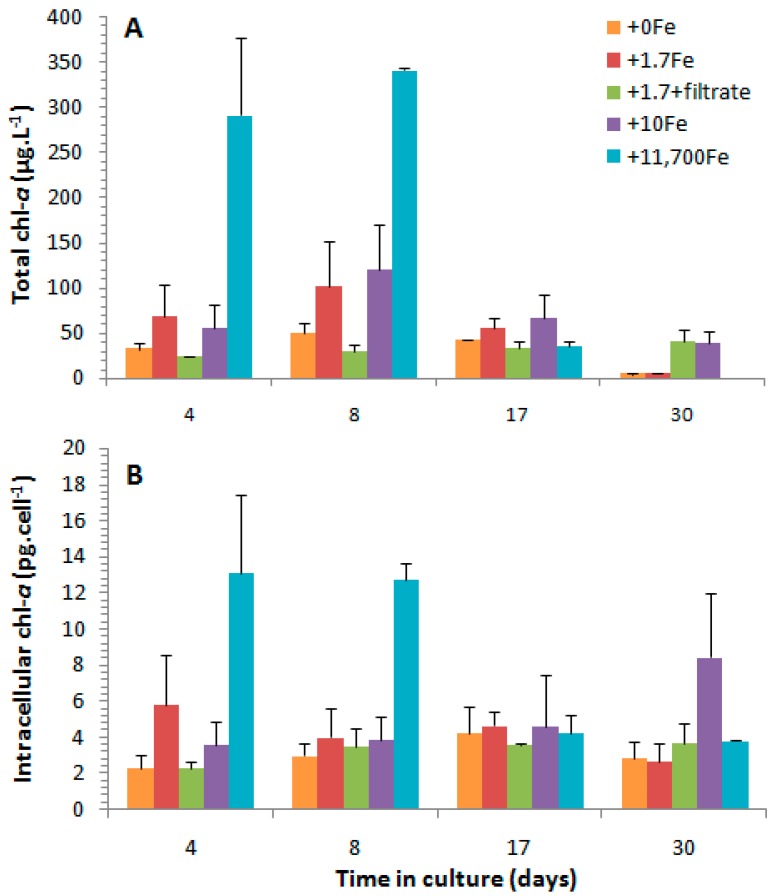
Total (**A**, μg·L^−1^) and intracellular (**B**, pg·cell^−1^) chlorophyll-*a* (chl-*a*) concentrations (average ± standard deviation, *n* = 2) throughout the growth cycle of *Bacillaria* sp. in cultures that received the addition of distinct iron concentrations (nmol·L^−1^): +0Fe; +1.7Fe; +10Fe; and +11,700Fe and the extra treatment (+1.7Fe+Psm) with the addition of 1.7 nmol·Fe·L^−1^ and the dissolved cell contents from a *P. multiseries* culture containing 52 ng DA·mL^−1^ and other undetermined compounds.

The average chlorophyll-*a* (chl-*a*) concentrations ranged from 0.35 to 341.2 µg·L^−1^ over the experimental period and across all treatments, with the highest values recorded in treatment +11,700Fe and the lowest ones in the treatment with no addition of Fe (Figure 5). Considering the two treatments that received the addition of the same iron concentration (1.7 nmol·L^−1^), +1.7Fe and +1.7Fe+Psm, chl-*a* concentrations were usually lower in the latter, in which *Bacillaria* sp. cells were also exposed to the dissolved compounds from a *P. multiseries* culture (see Supplementary Table S3 for statistical results). In general, chl-*a* concentrations followed the same temporal variation as cell density, attaining the maximum values at late exponential growth phase (day 8) and the minimum ones at the end of the experiment (day 30), except for treatment +1,7Fe+Psm, in which values increased at slower rates, but constantly over time (Figure 4 and Figure 5).

Considering the chl-*a* concentration on a per cell basis, intracellular concentrations were significantly higher in treatment +11,700Fe, attaining up to 12.7–13.1 pg·cell^−1^ during the exponential growth phase, and there were no significant differences among the average values measured in the other treatments (2.3–8.4 pg·cell^−1^) (Figure 5 and Table S2).

## 3. Discussion

### 3.1. Effects of Fe-Enrichment on the Growth of P. multiseries and Bacillaria *sp*.

The *P. multiseries* strain tested in this study reached similar cell densities and exponential growth rates in cultures exposed to different levels of Fe-enrichment. Similar results have been previously reported for a different *P. multiseries* isolate in cultures containing 0, 120, or 11,700 nmol·Fe·L^−1^ [24]. In both studies, *P. multiseries* exhibited no signs of growth limitation by Fe shortage in static cultures, even when no iron was added to the Fe-depleted medium. In our experiment, only very limited, trace amounts of Fe must have been available in the +0Fe treatment, transported both intra- and extra-cellularly with the inoculum, even after successive generations of culture acclimation under low concentrations of trace metals.

*P. multiseries* must, therefore, possess a very effective adaptation to limiting Fe conditions, as already indicated by Wells et al. [10] in another laboratory study. Accordingly, *Pseudo-nitzschia* spp. exhibited the most efficient physiological acclimation under low-Fe availability among the entire phytoplankton assemblage exposed to decreasing Fe concentrations in the Antarctic Peninsula [31].

Besides the lack of growth inhibition, there was no apparent effect of Fe availability on chain formation by *P. multiseries* cells in the present study. The length of linear chains of cells, i.e., the number of cells composing the chain, may vary according to the diatom species and be related to the turbulence and/or physiological state of the cells. As indicated by Lelong et al. [12], *P. multiseries* cells growing exponentially under replete nutrient conditions form longer chains, which become shorter and eventually detach into single cells as growth is limited during stationary phase. Thus, the similar incidence and length of *P. multiseries* stepped cell chains among the treatments in our experiment also indicate that the cultures were not growth-inhibited under any condition.

For *Bacillaria* sp., both the total chl-*a* concentrations (ng·mL^−1^) and the intracellular chl-*a* contents (pg·cell^−1^) were much higher in the cultures that received the greatest Fe addition (11,700 nmol·L^−1^), especially during the exponential growth phase. However, even though *Bacillaria* sp. cells had a lower photosynthetic capacity under lower concentrations of Fe, chain formation, as well as the maximum cell densities and growth rates, were surprisingly comparable among the experimental treatments. This indicates that, similar to *P. multiseries*, *Bacillaria* sp. also exhibited an efficient mechanism of adaptation to low Fe conditions.

The mechanisms and strategies explaining the efficient adaptation of *P. multiseries* and *Bacillaria* sp. to low Fe concentrations are not fully comprehended. Sarthou et al. [32] suggested that cell size reduction may provide diatoms with an adaptive advantage under limiting Fe concentrations. Reducing the Fe demand for cellular growth—and consequently the Fe:C ratio—may represent another strategy. In order to achieve that, diatoms might minimize the metabolic pathways that require greater Fe amounts, replace Fe-containing proteins with alternate ones containing different metals in their structures, or more efficiently use their Fe cell quotas [32]. During periods of high Fe availability, some coastal diatoms may store exceeding amounts of Fe at 20–30-fold greater Fe:C ratios than necessary for maximum cell growth [32]. Furthermore, certain pennate diatoms, including *Pseudo-nitzschia* species, contain ferritin, an Fe-concentrating protein that allows the cells to store Fe under periods of intermittent availability [33]. This protein is presumably related to the chloroplasts, regulating the distribution and storage of Fe, which would ultimately assure the proper functioning of the photosynthetic apparatus [34]. In addition to the above mentioned adaptive strategies, toxigenic *Pseudo-nitzschia* species may hypothetically use DA as a trace metal chelator. The fraction of toxin released into the water would, therefore, facilitate the acquisition of Fe at low concentrations [12], as further discussed in Section 3.2.

### 3.2. Effects of Fe-Enrichment on Toxin Production by P. multiseries

There was an increase in both intra- and extracellular DA concentrations during and after the stationary growth phase of our *P. multiseries* cultures. For the most toxigenic *Pseudo-nitzschia* species, including *P. multiseries*, an inverse relationship between toxin synthesis and cell division rate is usually reported over the growth cycle, with maximum intracellular and extracellular DA levels attained when growth is arrested at stationary and decline phases, respectively [12,35]. The occurrence of higher intracellular DA contents in our study at decline phase—and not stationary phase, as usual (e.g., [36])—may be due to a methodological artifact, since we only considered visibly living cells (i.e., with apparent chloroplasts) in our calculations of intracellular toxin levels.

In the present study, the highest intracellular DA concentrations were measured in the treatment that received the greatest addition of iron (+11,700 nmol·L^−1^). Similarly, in another culture study with *P. multiseries* [24], total DA production increased up to 10-fold in cultures with high-Fe concentrations (11,700 nmol·L^−1^) during the stationary growth phase, relative to low-Fe conditions (addition of 0–120 nmol·L^−1^). The authors postulated that Fe-stressed cells reduced their capacity to acquire nitrogen, which is essential for DA biosynthesis [24].

The levels of extracellular DA in our *P. multiseries* cultures, in contrast, were not consistently affected by the amount of iron added to the medium. The highest concentrations were measured in the treatment that received the greatest Fe addition during the stationary phase, and in the treatment with no Fe addition during the decline phase. Variations in the concentration of dissolved DA may result from an intricate balance between high photodegradation rates [37] and shifts in the rates of toxin release by the cells, which, in turn, are affected by differential cell death.

Maldonado et al. [25], in contrast, measured higher intra- and extracellular DA levels in Fe-limited cultures relative to an Fe-sufficient condition. According to the authors, DA production and release by *Pseudo-nitzschia* spp. could be a response to the stress caused by either lack of Fe or excess of Cu. The efficient adaptation of toxigenic *Pseudo-nitzschia* cells to Fe-limiting conditions would be therefore associated with the production and release of DA, which would be triggered under Fe privation [10]. Unfortunately, we could not reach the growth-limiting condition necessary to cause stress due to the lack of Fe in our study, even in the treatment with no addition of Fe.

The apparent contrasting results reported by Bates et al. [24] and Maldonado et al. [25] are not directly comparable though, since they measured the toxin concentrations in distinct culture fractions and were obtained from cultures at different growth phases (Table 3), as already noted by Lelong et al. (2012) [12]. In the present study, growth and DA production were evaluated over the entire growth cycle of a *P. multiseries* strain subjected to different Fe-enrichment conditions, and the toxin content was measured in both the particulate (intracellular) and dissolved (extracellular) culture fractions.

The highest concentrations of DA in the cultures that received the addition of the greatest Fe concentrations highlights that Fe is decidedly important for DA biosynthesis, as already noted by Bates et al. [24]. Likewise, Fe is essential to many metabolic processes that require electron transfer reactions, such as photosynthesis, respiration, and nitrogen assimilation [32].

### 3.3. Effects of the Cell Contents from P. multiseries Cultures on Bacillaria *sp*.

When the dissolved cell contents from a *P. multiseries* culture were added to one of the treatments which the diatom *Bacillaria* sp. was exposed to, there was a notable growth inhibition, as assessed by a very limited increase in both cell density and total chl-*a* concentration over the growth cycle. Such growth inhibition must have been caused by a reduction in Fe availability, or, most likely, due to the allelopathic effect of an undetermined harmful compound produced by *P. multiseries*.

No growth inhibition was reported when either *P. multiseries* or DA was mixed in batch cultures with different flagellated phytoplankton species (*Chrysochromulina ericina*, *Eutreptiella gymnastica, Karenia mikimotoi, Heterocapsa triquetra, Heterosigma akashiwo, Prorocentrum minimum*, *Prorocentrum micans, Pyramimonas propulsa*, and *Rhodomonas marina*) in a previous study [38]. However, the presence of allelopathic effects may be species-specific. In a recent study [39], *Pseudo-nitzschia pungens* limited the growth of *Akashiwo sanguinea*, *Chattonella marina*, *Phaeocystis globosa*, *Rhodomonas salina*, and *P. minimum,* and two different strains of *P. multiseries* affected negatively the growth of *A. sanguinea*. Considering the ecologically relevant cell densities tested, the observed allelopathic effect would represent a competitive advantage for *Pseudo-nitzschia* spp. in the field, playing an important role in the formation and persistence of both natural blooms and those following artificial fertilization with iron in high-nutrient, low-chlorophyll (HNLC) regions [39].

Allelopathic effects may be even more potent when the inhibited phytoplankton species is exposed to algal culture filtrates (i.e., cell-free culture medium) rather than to algal co-cultures (i.e., with allelopathic cells present). For instance, the filtered fraction from a *P. multiseries* culture has reduced 30–50% of the dinoflagellate *A. sanguinea* cell density relative to the control condition [39]. Moreover, in marine environments, the nutrient limitation can further increase the production of allelochemicals by certain phytoplankton species and/or strengthen their adverse effects over other algal species [40]. In the present study, under low iron concentration (+1.7 nmol·L^−1^ condition), the presence of dissolved compounds produced by *P. multiseries*, including 52 ng DA·mL^−1^ and other undetermined substances, reduced 50–70% of the cell density and 70–90% of the total chl-*a* content (ng·mL^−1^) of *Bacillaria* sp. at early stationary growth phase. To the best of our knowledge, this is the first report of allelopathic inhibition of a diatom species by DA-producing *Pseudo-nitzschia* at ecologically relevant cell densities (i.e., ~8700 cell equivalent·mL^−1^); massive blooms can reach up to ~13,000 cells·mL^−1^ and 20 ng of particulate DA·mL^−1^, for instance [5]. Moreover, although frequently found in plankton, *Bacillaria* spp. are typically benthic, which suggests a broad action mechanism for the allelopathic effect reported herein for *P. multiseries.*

## 4. Material and Methods

### 4.1. Culture Establishment and Acclimation

A toxic *Pseudo-nitzschia multiseries* strain, kindly provided by Monterey Bay Aquarium Research Institute—MBARI (Moss Landing, CA, USA), and a strain of *Bacillaria* sp. isolated from the Estuarine Complex of Paranaguá (25°30′ S, 48°25′ W), southern Brazil, were used in the present study.

Prior to the experiment, cultures of *P. multiseries* were maintained for seven generations (i.e., successive inoculations) in f/2 medium [41], modified to only 5% of the standard concentration of trace metals and for two additional generations with no addition of trace metals. Similarly, cultures of *Bacillaria* sp. were maintained for two generations in f/2 medium with no addition of trace metals, in order to reduce Fe carryover when the inoculum (17% of the total volume) was added to the experimental units, as described below.

### 4.2. Growth and Domoic Acid Production Experiments

Two similar, independent experiments were performed to assess the effects of increasing Fe concentrations on the growth and chain formation by *P. multiseries* (Experiment 1) and *Bacillaria* sp. (Experiment 2), as well as on DA production and release (Experiment 1 only) or pigment synthesis (Experiment 2 only). In addition, the effects of dissolved substances released by *P. multiseries* to the growth of an Fe-depleted *Bacillaria* sp. culture were also investigated in Experiment 2, as described below.

For both experiments, synthetic seawater was prepared using Ultrapure water (Mili-Q) and salts, according to the composition and concentrations of Aquil medium [42], and enriched with macronutrients following the f/2 formulation [41]. The artificial seawater was gently passed through glass columns filled with Chelex 100 ion exchange resin for removing eventual trace metals. Eighty-milliliter aliquots of the acclimated *P. multiseries* and *Bacillaria* sp. cultures in stationary growth phase were then added to 350 mL of the synthetic seawater and placed in 500 mL Erlenmeyer flasks. Each experimental unit was sterilized by microwave and cooled to room temperature. Subsequently, vitamins and EDTA-metal traces were added to the medium following the f/2 specifications, except for Fe, which was added as Fe-EDTA solution at different amounts according to each experimental treatment, as follows: 1.7 nmol·L^−1^, 10 nmol·L^−1^ and 11,700 nmol·L^−1^ (namely “+1.7Fe”, “+10Fe” and “+11,700Fe”, respectively). The last treatment corresponded to the standard concentration indicated for f/2 medium. Additionally, no iron was added to compose a fourth experimental treatment (“+0Fe”). Although precaution was taken to prevent contamination of the artificial seawater with Fe, it is possible that the inoculum carried traces of Fe, including intracellularly.

Four replicates were performed in individual flasks for each treatment of Experiment 1, and three replicates for Experiment 2. In the latter, an additional treatment (“+1.7Fe+Psm”) was obtained by passing 240 mL of non-axenic *P. multiseries* cultures under stationary growth (mean cell density: 46,700 cells·mL^−1^) through glass microfiber filters (25 mm diameter, 1.2 µm nominal porosity); then placing the filters into 20 mL of synthetic seawater and disrupting the retained cells with a ultrasound probe; re-filtering the solution with a 0.22 µm PTFE syringe filter to remove cell debris, and collecting the filtered fraction. A 6.66 mL aliquot of the filtered liquid was finally added to 343.34 mL of synthetic seawater, followed by the addition of 1.7 nmol Fe·L^−1^ and other nutrients at standard f/2 concentrations to compose each replicate flask of the +1.7Fe+Psm treatment. The resulting solution contained DA at 52 ng·mL^−1^ and other undetermined substances originally contained in 8680 *P. multiseries* cells·mL^−1^.

Experimental flasks were inoculated with 80 mL of homogenized, acclimated cultures of *P. multiseries* (Experiment 1) or *Bacillaria* sp. (Experiment 2) under early stationary growth. Experiments lasted for 30 days, during which static cultures were maintained in an illuminated microbiological incubator under the following controlled conditions: 18–19°C, 30–32 salinity, photoperiod 12:12 h, and irradiance ~140 μE·m^−2^·s^−1^. Samples (10 mL) were collected every 2–3 days throughout the growth curve and preserved with Lugol’s solution (1%). Cell density (cells·mL^−1^) and the proportion of cells forming linear chains were measured by light microscope counting using a Sedgewick–Rafter chamber. Additionally, 30 mL samples were collected at 4, 8, 17, and 30 days following the beginning of the experiment, and gently passed through Whatman^®^ GF/F glass microfiber filters (25 mm diameter, 0.7 µm particle retention) to determine the concentration of intra- and extracellular DA in *P. multiseries* or chlorophyll-*a* in *Bacillaria* sp. cultures.

### 4.3. Chemical Analysis

#### 4.3.1. Domoic Acid

Filters containing retained *P. multiseries* cells were exposed for 2 min to an ultrasound probe in order to break the cells and release the intracellular contents into 4 mL of 100% MeOH (J.T. Baker^®^, Trinidad and Tobago, 99.96% purity). The solution was then passed through syringe filters (Millipore^®^, 0.22 µm pore) to remove cell debris and the liquid was transferred to a vial for determination of the particulate (intracellular) DA concentration. In parallel, filtrate samples (30 mL) were loaded on a C–18 SEP-PAK cartridge and washed with 5 mL of 100% MeOH. The concentrated solution (6× concentration factor) was then recovered into a vial for determination of the dissolved (extracellular) DA concentration. Domoic acid quantification was performed by an external standard method with DA calibration solutions prepared by diluting certified reference material (IMB-NRC, Canada) in 100% MeOH at 5, 10, 50, 100, 250, and 300 ng·mL^−1^.

The DA analyses were performed by liquid chromatography coupled with ultraviolet detection (LC-UVD) in a Chromaster VWR Hitachi LC system attached to a photodiode 5430 detector, following the methodology described in Mafra et al. [43]. A RP–18 endcapped Purospher STAR C–18 column was used as solid phase and the mobile phase was composed of (A) water and (B) 100% acetonitrile, both containing 0.1% trifluoroacetic acid (TFA). Injections of 80 µL of freshly acidified samples (0.1% TFA) were performed in gradient elution mode (from 5 to 25% B in 25 min, holding for 2 min and back to 5% B in 3 min). Limits of detection (LD) and quantification (LQ) were statistically calculated from the formulas LD = [(3 * std)/b] and LQ = [10 * std/b], where “std” is the standard deviation of the peak area from repeated analysis (*n* = 5) of the minimum measured concentration of DA in a standard solution, and “b” is the slope of the calibration curve [43].

Only living cells (i.e., with visible chloroplasts) were considered for the calculation of cell density and intracellular DA concentration over time, while the sum of live and dead cells was used for the calculation of extracellular DA concentration on a per cell basis.

#### 4.3.2. Chlorophyll-*a*

Filters containing *Bacillaria* sp. cells were cut into small pieces and transferred to dark centrifuge tubes containing 4 mL of 90% acetone (Qhemis^®^, Jundiaí, SP, Brazil; HPLC/UV grade). Samples were maintained at −12 °C for 24 h and then centrifuged at 1000× *g* for 5 min. After attaining room temperature, 2 mL of the supernatant were transferred to a quartz cuvette and the chlorophyll-*a* fluorescence was determined in a Trilogy Laboratory fluorometer (Turner Designs^®^, San Jose, CA, USA) before and 90 s after the addition of 60 µL of 0.1 N HCl (Vetec^®^, Duque de Caxias, RJ, Brazil), in order to account for the concentration of pheophytin-*a* [44]. Chlorophyll-*a* was quantified from an external calibration curve made of successive dilutions of the analytical standard (Sigma-Aldrich^®^, Saint Louis, MO, USA) at 5167.0, 1033.4, 206.68, 41.34, and 8.27 µg·L^−1^.

### 4.4. Data Treatment and Statistical Analysis

Statistical analysis and plots were performed in R software [45]. A two-factor analysis of variance (ANOVA) was used to compare dissolved and particulate DA concentrations (extra- and intracellular fractions, respectively), among experimental treatments (“Tr”: fixed, four levels, +0Fe; +1.7Fe; +10Fe; +11,700Fe) and sampling times; (“Tm”: fixed, four levels, 4; 8; 17; 30 days, crossed with treatments) in Experiment 1, and among Tr (fixed, five levels, +0Fe; +1.7Fe; +10Fe; +11,700Fe; +1.7Fe+Psm) and Tm (fixed, four levels, 4; 8; 17; 30 days, crossed with treatments) in Experiment 2. For those terms in ANOVA that were found to be significant at *p* < 0.05, means were compared using Student–Newman–Keuls (SNK) tests. Normality and homoscedasticity assumptions were verified using Shapiro–Wilk and Cochran tests, respectively, and data were transformed to ln(x + 1) when necessary. ANOVAs and SNK tests were performed using package GAD [46] in R.

The maximum exponential growth rates (µ, d^−1^) were determined for Experiment 1 and Experiment 2 by the formula µ = [ln(n_1_) − ln(n_0_)]/(t_1_ − t_0_), where ln(n_1_) and ln(n_0_) are the natural logarithms of the cell densities (cell·mL^−1^) at the end (t_1_, d) and beginning (t_0_, d) of the exponential phase for each treatment. Additionally, in order to facilitate the comparison across all treatments, the exponential growth rate was also calculated between the days 0 and 8 (µ_0–8_), when cells were still multiplying exponentially in all cultures. Exponential growth rates (µ_0–8_ and µ) and cell densities from both Experiment 1 and Experiment 2 were individually compared among experimental treatments using a one-factor ANOVA. Assumptions checks and post-hoc comparisons of means were conducted as described for the statistical analysis on DA concentrations.

Generalized additive models (GAMs) were adjusted for *P. multiseries* and *Bacillaria* sp. cell densities using culture days and treatments as explanatory variables. Models were fitted using penalized cubic regression splines [47] in the mgcv package [38] of the R language. Two candidate models were adjusted: (i) one including the interaction between days (numerical predictor) and treatments (categorical predictor); and (ii) one without the interaction between days and treatments. Models were compared by an *F*-test and goodness of fit was assessed using Akaike Information Criteria (AIC). For both *P. multiseries* and *Bacillaria* sp. cell densities, the model that included the interaction was a better fit. Graphical diagnostics obtained with the gam.check() command in mgcv were used for model validation, in accordance with Zuur et al. [47].

## 5. Conclusions

The results from a series of laboratory experiments presented herein highlighted the great ability of two pennate diatoms, the domoic acid-producing *Pseudo-nitzschia multiseries* and the non-toxic *Bacillaria* sp., to survive and grow under low concentrations of iron. Moreover, the present study was the first to investigate the effects of different iron concentrations on the production and release of DA over the entire growth cycle of *Pseudo-nitzschia multiseries* in static cultures. Our results indicate that Fe is important for DA synthesis, since higher intracellular amounts of DA were detected in the cultures that received the addition of higher iron concentrations. Finally, the growth of *Bacillaria* sp. was inhibited by the presence of dissolved compounds originating from a *P. multiseries* culture, indicating, for the first time, an allelopathic effect of compounds released by *Pseudo-nitzschia* on another diatom species.

## Figures and Tables

**Table 1:** Exponential growth rate between days 0 and 8 of culture (μ_0–8_) and maximum growth rate (μ) (mean ± standard error) for *Pseudo-nitzschia multiseries* (*n* = 4) and *Bacillaria* sp. (*n* = 3), exposed to cultures that received the addition of distinct iron concentrations (nmol·L^−1^): +0Fe; +1.7Fe; +10Fe; and +11,700Fe. For *Bacillaria* sp., an extra treatment (+1.7Fe+Psm) was created with the addition of 1.7 nmol Fe·L^−1^ and the dissolved cell contents from a *P. multiseries* culture containing 52 ng DA·mL^−1^ and other undetermined compounds. DA: domoic acid.

Treatments	*P. multiseries*		*Bacillaria* sp.	
	µ_0–8_	µ	µ_0–8_	µ
+0Fe	0.25 (±0.05)	0.74 (±0.05)	0.28 (±0.02)	0.81 (±0.05)
+1.7Fe	0.28 (±0.02)	0.65 (±0.06)	0.30 (±0.01)	0.91 (±0.04)
+1.7Fe+Psm	N/A *	N/A	0.13 (±0.02)	0.36 (±0.03)
+10Fe	0.31 (±0.01)	0.54 (±0.03)	0.36 (±0.02)	0.80 (±0.16)
+11,700Fe	0.36 (±0.02)	0.71 (±0.09)	0.41 (±0.01)	1.25 (±0.02)

* N/A: not applicable.

**Table 2:** Average (± standard error; *n* = 4) intracellular (pg·cell^−1^), extracellular (pg·cell^−1^), and total (µg·mL^−1^) domoic acid (DA) concentration throughout the growth cycle of *Pseudo-nitzschia multiseries* in cultures that received the addition of distinct iron concentrations (nmol·L^−1^): +0Fe; +1.7Fe; +10Fe; and +11,700Fe. Only living cells were considered for the calculation of cell density and intracellular DA concentration, while the sum of live and dead cells was used for the calculation of extracellular DA concentration.

Days	Treatments	DA Concentration		
		Intracellular (pg·Cell^−1^)	Extracellular (pg·Cell^−1^)	Total (µg·mL^−1^)
4	+0Fe	0.04 (0.001)	0.44 (0.07)	0.01 (0.001)
	+1.7Fe	0.03 (0.001)	0.54 (0.09)	0.02 (0.002)
	+10Fe	0.04 (0.005)	0.72 (0.17)	0.02 (0.008)
	+11,700Fe	0.04 (0.005)	0.74 (0.20)	0.03 (0.007)
8	+0Fe	0.06 (0.01)	1.85 (1.02)	0.05 (0.02)
	+1.7Fe	0.06 (0.02)	0.81 (0.49)	0.03 (0.001)
	+10Fe	0.03 (0.006)	0.74 (0.18)	0.04 (0.01)
	+11,700Fe	0.20 (0.02)	0.55 (0.08)	0.04 (0.008)
17	+0Fe	0.26 (0.06)	1.95 (1.11)	0.10 (0.05)
	+1.7Fe	0.27 (0.07)	25.8 (16.7)	1.15 (0.65)
	+10Fe	0.14 (0.03)	2.15 (1.05)	0.18 (0.08)
	+11,700Fe	0.45 (0.11)	91.3 (38.5)	4.75 (1.88)
30	+0Fe	0.84 (0.31)	18.6 (11.5)	0.66 (0.34)
	+1.7Fe	1.21 (0.37)	4.56 (1.95)	0.20 (0.08)
	+10Fe	1.29 (0.42)	7.24 (5.50)	0.21 (0.14)
	+11,700Fe	2.07 (1.25)	6.83 (2.51)	0.28 (0.11)

**Table 3:** Summary of results obtained by this and previous studies on the particulate (P, intracellular), dissolved (D, extracellular), and total (T = P + D) domoic acid concentrations (pg·cell^−1^) produced by *Pseudo-nitzschia multiseries* during distinct growth phases in static cultures that received the addition of either low or high iron (Fe) concentrations.

	*Low-Fe Addition* (0–1.7 nmol·L^−1^)			*High-Fe Addition* (120–11,700 nmol·L^−1^)		
**Exponential phase**	**P**	**D**	**T**	**P**	**D**	**T**
Maldonado et al. (2002) [25]	0.07	1.8		0.03	0.21	
Present study	0.06	0.81–1.8		0.20	0.55	
**Early stationary phase**						
Bates et al. (2001) [24]			4.0			14–18
Present study	0.26	1.95–25.8		0.45	91.3	
**Declining/late stationary phase**						
Bates et al. (2001) [24]			5.0			22–46
Present study	0.84–1.21	4.56–18.6		2.07	6.83	

## Supplementary Materials

The following are available online at www.mdpi.com/1660-3397/15/10/331/s1, Table S1: Results of the analysis of variance (ANOVA) and SNK multiple comparison tests for cell density, growth rate between days 0 and 8 of culture (μ_0–8_) and maximum growth rate (μ) of *Pseudo-nitzschia multiseries* and *Bacillaria* sp., Table S2: Results of the analysis of variance (ANOVA) and SNK multiple comparison tests for intra- and extracellular domoic acid (DA) concentrations, Table S3: Results of the analysis of variance (ANOVA) and SNK multiple comparison tests for the total (µg·L^−1^) and intracellular (pg·cell^−1^) chlorophyll-*a* concentrations in *Bacillaria* sp. cultures.
