# Supplementary material for: Growth, Toxin Production and Allelopathic Effects of Pseudo-nitzschia multiseries under Iron-Enriched Conditions
Source: Mar Drugs. 2017 Oct 24;15(10):331. doi: 10.3390/md15100331 (PMC5666437; doi:10.3390/md15100331)
Supplement: Supplementary file 1 [file marinedrugs-15-00331-s001.pdf]

Table S1 Results of the analysis of variance (ANOVA) and SNK multiple comparison tests for cell density, growth rate between days 0 and 8 of culture (μ_{0-8}) and maximum growth rate (μ) of *Pseudo-nitzschia multiseries* and *Bacillaria* sp. Mean values were compared across treatments that received the addition of distinct iron (*Fe*) concentrations, in nmol.L^{-1} (Tr: *+0Fe*; *+1.7Fe*; *+10Fe*; *+11,700Fe*). For *Bacillaria* sp., an extra treatment *(+1.7Fe+Psm*) was created with the addition of 1.7 nmol Fe.L^{-1} and the dissolved cell content from a *P. multiseries* culture containing 52 ng DA.mL^{-1} and other undetermined compounds. Significant terms of interest ($\alpha = 0.05$) are highlighted in bold.

	P. multiseries **cell density**				***Bacillaria*** **sp. cell density**			
	df	MS	F	p	df	MS	F	p
Tr	3	233543906	3.0955	0.0675	4	70867500	6.3477	**0.0176**
Residual	12	75446719			7	11164286		
SNK test	–				+0Fe = +1.7Fe = +10Fe = +11,700Fe = +1.7Fe+Psm			
	P. multiseries **growth rate (μ_{0-8})**				***Bacillaria*** **sp. growth rate (μ_{0-8})**			
	df	MS	F	p	df	MS	F	p
Tr	3	0.0098	2.5812	0.1020	4	0.0237504	20.772	**0.0006**
Residual	12	0.0038			7	0.0011434		
SNK test	–				+0Fe = +1.7Fe = +10Fe = +11,700Fe > +1.7Fe+Psm			
	P. multiseries **maximum growth rate (μ)**				***Bacillaria*** **sp. maximum growth rate (μ)**			
	df	MS	F	p	df	MS	F	p
Tr	3	0.0303	2.0327	0.1631	4	0.1992	8.057	**0.0093**
Residual	12	0.0149			7	0.0247		
SNK test	–				+0Fe = +1.7Fe = +10Fe = +11,700Fe > +1.7Fe+Psm			

Table S2 Results of the analysis of variance (ANOVA) and SNK multiple comparison tests for intra- and extracellular domoic acid (DA) concentrations. Mean values were compared across treatments that received the addition of distinct iron (*Fe*) concentrations, in nmol.L^{-1} (Tr: *+0Fe*; *+1.7Fe*; *+10Fe*; *+11,700Fe*), sampling times (Tm: 4; 8; 17; 30 d), and the interaction between them. Significant terms of interest (α = 0.05) are highlighted in bold.

		**DA (pg.cell^{-1})**							
		Intracellular				Extracellular			
	Df	MS	F	p	SNK	MS	F	p	SNK
Tr	3	1.89	**6.66**	**7.5×10^{-4}**	*+10Fe=+1.7Fe=+0Fe<+11,700Fe*	1.91	1.009	0.39	N/A*
Tm	3	33.1	**116.6**	**2.2×10^{-16}**	4<8<17<30	41.08	**21.60**	**5.4×10^{-9}**	8=4=30<17
Tr×Tm	9	0.48	1.70	0.11	N/A*	3.18	1.67	0.12	N/A*

* N/A: not applicable.

Table S3 Results of the analysis of variance (ANOVA) and SNK multiple comparison tests for the total (µg.L^{-1}) and intracellular (pg.cell^{-1}) chlorophyll-*a* concentrations in *Bacillaria* sp. cultures. Mean values were compared across treatments that received the addition of distinct iron (*Fe*) concentrations (nmol.L^{-1}), or iron and the dissolved cell content from a *P. multiseries* (*Psm*) culture (Tr: *+0*; *+1.7*; *+10*; *+11,700*; and *+1.7+Psm*), sampling times (Tm: 4; 8; 17; 30 d), and the interaction between them. Significant terms of interest (α = 0.05) are highlighted in bold. See methods for details.

		**Total chlorophyll-*a* (µg.L^{-1})**				**Intracellular chlorophyll-*a* (pg.cell^{-1})**			
	**Df**	**MS**	**F**	**P**	**SNK**	**MS**	**F**	**P**	**SNK**
**Tr**	4	0.55	4.19	0.012		0.86	3.30	0.03	
**Tm**	3	9.27	70.6	8.1x10^{-11}		0.41	1.57	0.22	
**Tr×Tm**	12	2.30	**17.5**	**4.8x10^{-8}**	Day 04: *+1.7+Psm=+0=+10<+1.7<+11,700*<br>Day 08: *+1.7+Psm<+0<+10=+1.7<+11,700*<br>Day 17: *+1.7+Psm=+11,700=+0=+10=+1,7*<br>Day 30: *+11,700<+0=+1,7<+10=+1.7+Psm*	0.86	**3.33**	**8.5x10^{-3}**	Day 04: *+0=+1.7+Psm=+10<+1.7<+11,700*<br>Day 08: *+0=+10=+1.7+Psm=+1.7<+11,700*<br>Day 17: *+10=+1.7+Psm=+0=+11,700=+1.7*<br>Day 30: *+11,700<+1.7=+0=+1.7+Psm<+10*
